# Supplementary material for: In-flight positional and energy use data set of a DJI Matrice 100 quadcopter for small package delivery
Source: Sci Data. 2021 Jun 18;8:155. doi: 10.1038/s41597-021-00930-x (PMC8213793; doi:10.1038/s41597-021-00930-x)
Supplement: Supplementary file 1 — Supplementary Information [file 41597_2021_930_MOESM1_ESM.pdf]

In-flight positional and energy use data set of a DJI Matrice 100 quadcopter for small package delivery: Supplementary information

Checklists

Pre-Flight

- Inspect the aircraft
  - Visually inspect the condition of the unmanned aircraft system components
  - Inspect the airframe structure, including undercarriage, all flight control surfaces and linkages
  - Inspect registration markings for proper display and legibility
  - Inspect moveable control surface(s), including airframe attachment point(s)
  - Inspect servo motor(s), including attachment point(s)
  - Inspect the propulsion system, including powerplant(s), propeller(s), rotor(s), ducted fan(s), etc.
- Verify all systems (e.g. aircraft, control unit) have an adequate energy supply for the intended operation and are functioning properly
- Inspect the avionics, including control link transceiver, communication/navigation equipment and antenna(s)
- Check UAS compass and calibrate UAS compass prior to any flight if necessary.
- Check that the display panel, if used, is functioning properly
- Check ground support equipment, including takeoff and landing systems, for proper operation
- Check on board navigation and communication data links
- Check flight termination system and manual override switch
- Check fuel for correct type and quantity
- Check battery levels for the aircraft and control station
- Check that the payload is securely attached
- Verify communication with UAS and that the UAS has acquired GPS location from at least 4 satellites
- Arm and disarm the UAS propellers to inspect for any imbalance or irregular operation
- If required by flight path walk through, verify any noted obstructions that may interfere with the UAS
- At a controlled low altitude, fly manually within range of any interference and recheck all controls and stability

Post-Flight

- Wait for all motors and rotors to stop
- Verify stop sensor recording
- Disconnect the battery
- Inspect the airframe for any in-flight damage
- Check wiring is secure
- Check connectors are fully connected

- Motors are securely mounted
- Propellers are securely fastened
- Propellers rotate freely without any obstructions (e.g. hitting edges of incorrectly fitted indoor hull) or have been removed.
- All sensors are unobstructed and undamaged

Anemometer Magnitude Bias and Ego Motion Correction

One way to remove the magnitude bias and ego motion in the wind measurements is by using the inertial velocity data and the data collected by Brushi et al. [1]of a quadrotor flying with a ultrasonic wind sensor inside a wind tunnel. Given the similarity between our setup and theirs, the wind-tunnel results[1] are used to construct a polynomial mapping between measured and actual wind speeds. As the data lacks points in the lower range of values, it is augmented with results from our own setup. The data-points and the curve fit are plotted in Figure S1. The UAV was flown in hover and constant ground speed mode for an extended amount of time in near-zero wind conditions to get the lower range of values. The magnitude correction polynomial and ego-motion correction is given in Equation S1.

$$\begin{aligned} w_m^{[actual]} &= -0.002(w_m^{[raw]})^4 + 0.052(w_m^{[raw]})^3 - 0.421(w_m^{[raw]})^2 + 1.917 w_m^{[raw]} - 2.7 \\ w_N &= (w_m^{[actual]})\cos(w_\theta^{[raw]}) - V_N \\ w_E &= (w_m^{[actual]})\sin(w_\theta^{[raw]}) - V_E \\ w_m^{[corrected]} &= \sqrt{w_N^2 + w_E^2} \\ w_\theta^{[corrected]} &= \arctan(w_E, w_N) \end{aligned} \tag{S1}$$

where V_N and V_E represent the inertial speed of the UAV in North and East directions, and w_N and w_E represent the corrected wind components in North and East directions.

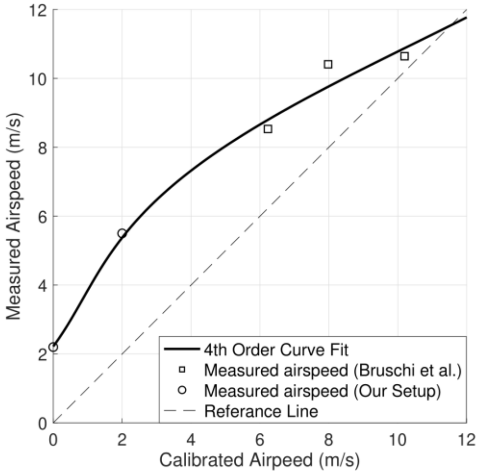

Figure S1 *Anemometer corrections*

The data set contains the raw measurements and is not modified with these proposed methods, as the focus of this work is not about obtaining accurate wind field estimations. The area of wind estimation through the use of a drone is an ongoing field of research[2], as the rotors induce complex air disturbances which affect the anemometer. Thus, we left our data in a state where various current or future methods of wind correction can be applied if needed.

References

1. Bruschi, P., Piotto, M., Dell'Agnello, F., Ware, J. & Roy, N. Wind speed and direction detection by means of solid-state anemometers embedded on small quadcopters. *Procedia Eng.* **168**, 802–805 (2016).

2. Patrikar, J., Moon, B. & Scherer, S. Wind and the City: Utilizing UAV-Based In-Situ Measurements for Estimating Urban Wind Fields. in *Proceedings of (IROS) IEEE/RSJ International Conference on Intelligent Robots and Systems* (IEEE/RSJ, 2020).
